# Using caputo-fabrizio derivative for the transmission of mathematical model epidemic Corona Virus

**DOI:** 10.1007/s40324-020-00230-1

**Published:** 2020-09-26

**Authors:** M. Tahir, G. Zaman, S. I. A Shah

**Affiliations:** 1 Department Of Mathematics, Northern University, Nowshera, KPK Pakistan; 2 Department of Mathematics, University of Malakand, Chakdara District Lower Dir, KPK Pakistan; 3 Department Of Mathematics, Islamia College University, 25000 Peshawar, Pakistan

**Keywords:** Reservoir to person population(RP), Visitors population(VP), Mathematical model, $R_0$, Caputo-Fabrizio derivative, Numerical Simulation.

## Abstract

Just in a week a rapidly spreading corona virus which was originated in Wuhan, city of China, infected more than 20,000 people and also killed at least 427 people in that week worldwide. Corona virus is transmissible and spreading from person to person, while the Chinese commanded authorities are scrambling to treat a flood of new patients in Chines successfully. The said Corona virus has been spread from an initial outbreak in Wuhan, city of China, and invade 25 other worldwide countries. In this article, we considered the mathematical model (Chen et al. Infect Dis Poverty, https://doi.org/10.1186/s40249-020-00640-3) in which Bats-Hosts-Reservoir-People and their transmission was taken, while we introduced the population of susceptible Bats and visitors to Wuhan city or any country in same mathematical model. Now we studying two types of populations first Bats-Hosts-Reservoir-People (Chen et al. Infect Dis Poverty, https://doi.org/10.1186/s40249-020-00640-3, also introducing susceptible Bats and second visitors to Wuhan city, china or any country in the same model. We used Caputo-Fabrizio derivative with provided result that the addition of susceptible Bats and visitors are not responsible in spread of infection. The numerical result also supported our model.

## Background

The people of the entire world seen a very new epidemic thread in end of 2019 and 2020. After the middle East Respiratory Syndrome(MERS), in middle east, a new virus Corona attacked the “Wuhan city, China”. The Corona virus was first emerged in late December and has killed more than 2500 people there, this means “Wuhan”, China alone accounted for nearly 80 percent of the country’s with total deaths occur 3299 or more. Before this, the World Health Organization (WHO) China Country Office informed in 31, December 2019 about pneumonia(unknown cause) detected in “Wuhan” city, Hubei China and WHO announced novel Corona virus (2019-nCoV). The International Committee of Taxonomy assigned it severe acute respiratory syndrome Corona virus-2(SARS-CoV-2) on 11 February, 2020 [12, 31, 34]. Similarly some of the best approaches was studied and presented in [4, 15, 18, 35]. All the country then infected, and an perception was drawn that visitors involve in this transmission of new virus mostly.

Scientists of all the world then felt an urgent mathematical model to estimate the transmission of this disease in China. Several researches focusing and developed mathematical models for MERS [21, 30, 32]. In these mathematical model the scientists trying the reproductive number which responsible for whole model [10, 23, 32]. The people are awarded from different methods, and control strategies to make possible precautions against Corona virus. Here the main aim is to estimate positivity, equilibrium, and Boundedness of these models [11, 13, 16, 27]. The recent and new ideas was also discussed in [1–3, 5, 14, 19, 25, 26]. While one of the best approach towards mathematical models is optimal control [17, 20, 22, 29]. Many different methods are adopted for optimal purpose. One best attempt was done in [31].

In our work we try to vanish and prove the wrong perception that susceptible Bats and visitors are the spreading agent of Corona virus in “wuhan” or any other country. Here we focus on the study done in [9] which was Bats-Hosts-Reservoir-People(BHRP) and its different transmission ways of Corona virus in any population. We just interesting in human-to-human transmission, so for that issue we introduced Bats population and visitors population who visit Wuhan China or any other country. We developed a mathematical model for the transmission of Corona virus Reservoir-People-Visitors(RPV) with introducing susceptible bats and estimate the value of $R_0$. We apply Caputo-Fabrizio derivative approach towards this issue, and a numerical simulation in the last.

## Data source

The named Corona virus which was COVID-19 which was SARS-CoV-2 in the first and the model was taken from the published literature [21]. The epidemic and its sharp curve was considered from 7 December, 2019 to 31 March, 2020 is collected for this study, while simulation step size is 1 week.

## Simulation methods and statistical analysis

For simulation and curve fitting we have used Runge-Kutta-fourth-order method. The reproductive number for first model was considered 0.5 and also we considered $R_0$ is equal to 1 for Bats and visitors model.

## Mathematical model and transmission of Bats-Hosts-Reservoir-People(BHRP)

In 19 January, 2020 [8] published BHRP mathematical transmission model in bioRxiv, with the following certain assumptions:

**Bats population:** 1:) Divided into four section, susceptible, exposed, infected, and removed are $$(S_B),(E_B),(I_B)$$ and $$(R_B)$$ respectively. But in our model we considered susceptible Bats $$(S_B)$$ only, and ignored the remaining because of no concern in human population. While $$n_B$$ and $$m_B$$ are taken the birth and death rate. $$ _B$$ is the rate of infection from $$S_B$$ when contact with $$I_B$$.

**Host Population:** 2:) Population of host is also divided in four compartments: $(S_H), (E_H), (I_H)$ and $(R_H)$ denoted susceptible, exposed, infected, and removed hosts. Here we leave all the compartments of the Hosts population, due to no role in human population infection.

**Reservoir Population:** Here *W* denoted SARS-CoV-2 in reservoir (the seafood market). In our study the rate of asymptomatic infected people and symptomatic infected people export virus with $\mu _p$ and $\hat{\mu }_p$ respectively from markets or any other source.

**People Population:** This population is divided in to five classes, there five compartments are: $(S_p), (E_p), (I_p), (A_p)$ and $(R_p)$ denoted susceptible, exposed, symptomatic infected, asymptomatic infected, and removed peoples compartments. Here $m_p$ represent death rate. $\delta _p$ was defined for the proportion of asymptomatic infection, also the $S_p$ infected from *W* and $I_p$ with the transmission rate $\beta _W$ and $\beta _p$.

## The new bats and visitors model

Here we drawn some more assumptions for our model:

### Assumption 1

In our study we considered the Bats population only.

### Assumption 2

We ignored the transmission of Bats-Host population due to unknown spreading source.

### Assumption 3

Hosts population of Bats is totally ignored in this study because of its unknown relation and infection.

### Assumption 4

We leave other related terms and contact in reservoir population in this article.

### Assumption 5

We taken the class of susceptible Bats to check weather this class spread any infection in the concern model.

### Assumption 6

In this study we introduced visitors population compartment separately.

During in any outbreak the death rate is normally high, therefore, the model BHRP converted into BRP and then in BRV(Bats-reservoir-visitors) model by the following,

1 $$\begin{aligned} S_B^\bullet= & {} A_B-m_BS_B-\beta _BS_BI_B,\nonumber \\ S_p^\bullet= & {} A_B-m_BS_B-\beta _BS_B(I_P+\kappa A_p)-\beta _WS_pW,\nonumber \\ E_p^\bullet= & {} \beta _BS_B(I_P+\kappa A_p)+\beta _WS_pW-(1-\delta _p)W_pE_p-\delta _pW_pE_p-m_pE_p,\nonumber \\ I_p^\bullet= & {} (1-\delta _p)W_pE_p-(\gamma _p+m_p)I_p,\nonumber \\ A_p^\bullet= & {} \delta _pW_p^\prime E_p-(\gamma _p^\prime +m_p)A_p,\nonumber \\ R_p^\bullet= & {} \gamma _pI_p+\gamma _p^\prime A_p-m_pR_p,\nonumber \\ W^\bullet= & {} \mu _pI_p+\mu _p^\prime A_p-\epsilon W. \end{aligned}$$

In outbreak the visitors and hosts interaction was very slow in “Wuhan” or other country. Now introducing dynamic population of the visitors to any city or country is as in separate for estimation of infection,

2 $$\begin{aligned} S^\bullet= & {} \mu _N-\sigma (t)SI-\alpha S,\nonumber \\ E^\bullet= & {} \sigma (t)SI-\lambda E-\alpha E,\nonumber \\ I^\bullet= & {} \lambda E-\eta I-\alpha I. \end{aligned}$$

## Transmissibility of Corona Virus or SARS-CoV-2 based on the BRP And Bats-visitors model

In article, $R_0$ is assessed for transmissibility of Corona virus(SARS-CoV-2). While the, $R_0$ we defined as, the expected number of secondary infections with introducing any single infected individual to susceptible population [7, 29, 36]. The value of $R_0$ having two faces that is, $R_0 > 1$, or $R_0 < 1$, showing different characteristics of the model in any outbreak to control or for no out control. In this study, $R_0$ was deduced from the BRP model and Bats-visitors population by next generation matrix approach [12].

## Some results

Here in this subsection of study, we given some basic definition of the fractional calculus, which we used later,

### Definition A

Let $H\varepsilon G^1(c,d)$ and *d* is greater then *c*, also $\tau \varepsilon [0,1]$ then CFF derivative [6] is given as,

$$\begin{aligned} P^\tau _t(h(t))=\frac{M(\tau )}{(1-\tau )}\int ^\tau _0 \hat{h}(x)e^{-\tau (\frac{\tau -x}{1-\tau })}dx. \end{aligned}$$

Here $$M(\tau )$$, which implies normality with $$M(0)=M(1)=1$$ [6]. But if *H* is not contain in $$ G^1(c,d)$$ then we get,

$$\begin{aligned} P^\tau _t(h(t))=\frac{\tau M(\tau )}{(1-\tau )}\int ^\tau _a (h(t)-h(x))e^{-\tau (\frac{\tau -x}{1-\tau })}dx. \end{aligned}$$

### Definition B

When $\upsilon =\frac{(1-\tau )}{\tau }\varepsilon [0,\infty )$ and $\tau =\frac{1}{1+\upsilon }\varepsilon [0,1]$ we have the following result,

$$\begin{aligned} P^\tau _t(h(t))=\frac{K(\upsilon )}{\upsilon }\int ^t_a\hat{h}(x)e^{(-\frac{t-x}{\upsilon })}dx. \end{aligned}$$

With

$$\begin{aligned} K(0)=K(\infty )=1. \end{aligned}$$

Applying $\lim \upsilon \rightarrow 0$ we get,

$$\begin{aligned} P^\tau _t(h(t))=\lim _{\upsilon \rightarrow 0} \frac{1}{\upsilon }e^{(-\frac{t-x}{\upsilon })}dx=\upsilon (x-t). \end{aligned}$$

This integral definition was provided by Losada and J. Nieto [24].

### Definition C

Suppose that $``0<\tau <1$” then CFFD integral of given function *h* is as,

3 $$\begin{aligned} I^\tau _th(t)=\frac{2(1-\tau )}{(2-\tau ) M(\tau )}h(\tau )+\frac{2\tau }{(2-\tau ) M(\tau ))}\int ^t_0h(s)ds,t\ge 0. \end{aligned}$$

The above equation will also be written in the following as,

$$\begin{aligned} \frac{2(1-\tau )}{(2-\tau ) M(\tau )}+\frac{2\tau }{(2-\tau ) M(\tau ))}=1. \end{aligned}$$

This gives us that $M(\tau )=\frac{2}{(2-\tau )}$, with $0<\tau <1$. From Eq. (3) a new form of the above $``Caputo$ $-$ $Fabrizio$ *fractinal* *derivative* of order $0<\tau <1$ which was further investigated by Losada and J. Nieto [24].

$$\begin{aligned} P^\tau _th(t)=\frac{1}{(1-\tau )}\int ^t_0\hat{h}(x)e^{(\tau \frac{(t-x)}{(1-\tau )})}dx. \end{aligned}$$

We use the above form in mathematical model of HBV and diabetes model [28, 33].

## Formulation of Model BVP from BRPV

Now we are going to replace system (1) by the new CF fractional derivative, as,

4 $$\begin{aligned} C_0FD^\tau _tS_B= & {} A_B-m_BS_B-\beta _BS_BI_B,\nonumber \\ C_0FD^\tau _tS_p= & {} A_B-m_BS_B-\beta _BS_B(I_P+\kappa A_p)-\beta _WS_pW,\nonumber \\ C_0FD^\tau _tE_p= & {} \beta _BS_B(I_P+\kappa A_p)+\beta _WS_pW-(1-\delta _p)W_pE_p-\delta _pW_pE_p-m_pE_p,\nonumber \\ C_0FD^\tau _tI_p= & {} (1-\delta _p)W_pE_p-(\gamma _p+m_p)I_p,\nonumber \\ C_0FD^\tau _tA_p= & {} \delta _pW_p^\prime E_p-(\gamma _p^\prime +m_p)A_p,\nonumber \\ C_0FD^\tau _tR_p= & {} \gamma _pI_p+\gamma _p^\prime A_p-m_pR_p,\nonumber \\ C_0FD^\tau _tW= & {} \mu _pI_p+\mu _p^\prime A_p-\epsilon W. \end{aligned}$$

Similarly we also replace Eq. (2) by CF fractional derivative by below,

5 $$\begin{aligned} C_0FD^\tau _tS= & {} \mu _N-\sigma (t)SI-\alpha S,\nonumber \\ C_0FD^\tau _tE= & {} \sigma (t)SI-\lambda E-\alpha E,\nonumber \\ C_0FD^\tau _tE= & {} \lambda E-\eta I-\alpha I. \end{aligned}$$

## Basic reproductive number

The calculate the basic reproductive number for system (1) is as,

$$\begin{aligned} R_0=\frac{A_B(1-\delta _p)W_p+(\gamma _pI_p)A_p}{\mu _p+\mu ^,_pA_p-W}+\frac{\beta _WA_p}{(\gamma ^,_p+m_p)}. \end{aligned}$$

## Methodology for our model

For existence for the model we use Fixed point theory. Now Caputo-Fabrizio fractional derivative for system (1), is,

6 $$\begin{aligned} S_B(t)-S_B(0)= & {} C_0FI^\tau _t\{A_B-m_BS_B-\beta _BS_BI_B\},\nonumber \\ S_p(t)-S_p(0)= & {} C_0FI^\tau _t\{A_B-m_BS_B-\beta _BS_B(I_P+\kappa A_p)-\beta _WS_pW\},\nonumber \\ E_p(t)-E_p(0)= & {} C_0FI^\tau _t\{\beta _BS_B(I_P+\kappa A_p)\nonumber \\&+\beta _WS_pW-(1-\delta _p)W_pE_p-\delta _pW_pE_p-m_pE_p\},\nonumber \\ I_p(t)-I_p(0)= & {} C_0FI^\tau _t\{(1-\delta _p)W_pE_p-(\gamma _p+m_p)I_p\},\nonumber \\ A_p(t)-A_p(0)= & {} C_0FI^\tau _t\{\delta _pW_p^\prime E_p-(\gamma _p^\prime +m_p)A_p\},\nonumber \\ R_p(t)-R_p(0)= & {} C_0FI^\tau _t\{\gamma _pI_p+\gamma _p^\prime A_p-m_pR_p\},\nonumber \\ W(t)-W(0)= & {} C_0FI^\tau _t\{\mu _pI_p+\mu _p^\prime A_p-\epsilon W\}. \end{aligned}$$

Similarly using Caputo-Fabrizio fractional derivative for Eq. (4), is in below,

7 $$\begin{aligned} S_(t)-S_(0)= & {} C_0FI^\tau _t\{\mu _N-\sigma (t)SI-\alpha S\},\nonumber \\ E_(t)-E_(0)= & {} C_0FI^\tau _t\{\sigma (t)SI-\lambda E-\alpha E\},\nonumber \\ I_(t)-I_(0)= & {} C_0FI^\tau _t\{\lambda E-\eta I-\alpha I\}. \end{aligned}$$

Now we applying the idea which used in [24] on Eqs. (6), and (7), while we get Eqs. (8) and (9), from Eqs. (6), and (7),

8 $$\begin{aligned} S_B(t)-S_B(0)= & {} \frac{2(1-\tau )}{(2-\tau )M(\tau )}\{A_B-m_BS_B-\beta _BS_BI_B\} \nonumber \\&\quad +&\frac{2\tau }{(2-\tau )M(\tau )} \int ^t_0\{A_B-m_BS_B-\beta _BS_BI_B\}dy,\nonumber \\ S_p(t)-S_p(0)= & {} \frac{2(1-\tau )}{(2-\tau )M(\tau )}\{A_B-m_BS_B-\beta _BS_B(I_P+\kappa A_p)-\beta _WS_pW\} \int ^t_0\{A_B-m_BS_B \nonumber \\&\quad -\beta _BS_B(I_P+\kappa A_p)-\beta _WS_pW\}dy,\nonumber \\ E_p(t)-E_p(0)= & {} \frac{2(1-\tau )}{(2-\tau )M(\tau )}\{\beta _BS_B(I_P+\kappa A_p)+\beta _WS_pW \nonumber \\&\quad -(1-\delta _p)W_pE_p-\delta _pW_pE_p-m_pE_p\} \int ^t_0\{A_B-m_BS_B \nonumber \\&\quad -\beta _BS_B(I_P+\kappa A_p)+\beta _WS_pW-(1-\delta _p)W_pE_p-\delta _pW_pE_p-m_pE_p\}dy,\nonumber \\ I_p(t)-I_p(0)= & {} \frac{2(1-\tau )}{(2-\tau )M(\tau )}\{1-\delta _p)W_pE_p-(\gamma _p+m_p)I_p\} \nonumber \\&\quad +\frac{2\tau }{(2-\tau )M(\tau )} \int ^t_0\{1-\delta _p)W_pE_p-(\gamma _p+m_p)I_p\}dy,\nonumber \\ A_p(t)-A_p(0)= & {} \frac{2(1-\tau )}{(2-\tau )M(\tau )}\{\delta _pW_p^\prime E_p-(\gamma _p^\prime +m_p)A_p\} \nonumber \\&\quad +\frac{2\tau }{(2-\tau )M(\tau )} \int ^t_0\{\delta _pW_p^\prime E_p-(\gamma _p^\prime +m_p)A_p\}dy,\nonumber \\ R_p(t)-R_p(0)= & {} \frac{2(1-\tau )}{(2-\tau )M(\tau )}\{\gamma _pI_p+\gamma _p^\prime A_p-m_pR_p\} \nonumber \\&\quad +\frac{2\tau }{(2-\tau )M(\tau )} \int ^t_0\{\gamma _pI_p+\gamma _p^\prime A_p-m_pR_p\}dy,\nonumber \\ W(t)-W(0)= & {} \frac{2(1-\tau )}{(2-\tau )M(\tau )}\{\mu _pI_p+\mu _p^\prime A_p-\epsilon W\} \nonumber \\&\quad +\frac{2\tau }{(2-\tau )M(\tau )} \int ^t_0\{\mu _pI_p+\mu _p^\prime A_p-\epsilon W\}dy. \end{aligned}$$

Here we applying the new idea of Losada and J.Nieto [24] on the system given in above, and we get,

9 $$\begin{aligned} S_(t)-S_(0)= & {} \frac{2(1-\tau )}{(2-\tau )M(\tau )}\{\mu _N-\sigma (t)SI-\alpha S\}+\frac{2\tau }{(2-\tau )M(\tau )} \int ^t_0\{\mu _N-\sigma (t)SI-\alpha S\},\nonumber \\ E_(t)-E_(0)= & {} \frac{2(1-\tau )}{(2-\tau )M(\tau )}\{\sigma (t)SI-\lambda E-\alpha E\} \nonumber \\&\quad +\frac{2\tau }{(2-\tau )M(\tau )} \int ^t_0\{\sigma (t)SI-\lambda E-\alpha E\}dy,\nonumber \\ S_B(t)-S_B(0)= & {} \frac{2(1-\tau )}{(2-\tau )M(\tau )}\{\lambda E-\eta I-\alpha I\} \nonumber \\&\quad +\frac{2\tau }{(2-\tau )M(\tau )} \int ^t_0\{\lambda E-\eta I-\alpha I\}dy. \end{aligned}$$

Now the simplified form of the Eqs. (10) and (11), after Eqs. (8) and (9), is below,

10 $$\begin{aligned} \Phi _1(t,S_B)= & {} \{A_B-m_BS_B-\beta _BS_BI_B\},\nonumber \\ \Phi _2(t,S_p)= & {} \{A_B-m_BS_B-\beta _BS_B(I_P+\kappa A_p)-\beta _WS_pW\},\nonumber \\ \Phi _3(t,E_p)= & {} \{\beta _BS_B(I_P+\kappa A_p)+\beta _WS_pW-(1-\delta _p)W_pE_p-\delta _pW_pE_p-m_pE_p\},\nonumber \\ \Phi _4(t,I_p)= & {} \{(1-\delta _p)W_pE_p-(\gamma _p+m_p)I_p\},\nonumber \\ \Phi _5(t,A_p)= & {} \{\delta _pW_p^\prime E_p-(\gamma _p^\prime +m_p)A_p\},\nonumber \\ \Phi _6(t,R_p)= & {} \{\gamma _pI_p+\gamma _p^\prime A_p-m_pR_p\},\nonumber \\ \Phi _7(t,W)= & {} \{\mu _pI_p+\mu _p^\prime A_p-\epsilon W\}. \end{aligned}$$

11 $$\begin{aligned} \prod _1(t,S)= & {} \{\mu _N-\sigma (t)SI-\alpha S\},\nonumber \\ \prod _2(t,S)= & {} \{\sigma (t)SI-\lambda E-\alpha E\},\nonumber \\ \prod _3(t,S)= & {} \{\lambda E-\eta I-\alpha I\}. \end{aligned}$$

### Theorem 10.1

The Kernals of $\Phi _1,\Phi _2,\Phi _3,\Phi _4,\Phi _5, \Phi _6$ and $\Phi _7$ fulfill the Lipschitz condition and contraction if the following inequality hold. $0\le (m_B+\beta _m\psi )e+I_B<1$, where $\psi =1$ and $e=\frac{1}{2}$.

### Proof

We prove the theorem for $\Phi _1,\Phi _2,\Phi _3,\Phi _4,\Phi _5, \Phi _6$ and $\Phi _7$ respectively. First suppose that *S* and $S_1$ are two function for $\Phi _1$, then,

12 $$\begin{aligned} \parallel \Phi _1(t,S_B)-\Phi _1(t,S_{1B})\parallel =\parallel -m_BS_B\{S_B(t)-S_B(t_1)\}-\beta _BI_B \{S_B(t)-S_B(t_1)\}\parallel . \end{aligned}$$

For Eq. (12) we apply triangle in-equality, we get,

13 $$\begin{aligned} \parallel \Phi _1(t,S_B)- & {} \Phi _1(t,S_{1B})\parallel \le \parallel -m_BS_B\{S_B(t)-S_B(t_1)\}\parallel \nonumber \\+ & {} \parallel \{- \beta _BI_B \{S_B(t)-S_B(t_1)\}\}\parallel .\nonumber \\\le & {} \parallel m_B\parallel +\parallel \{ \beta _B\parallel \parallel I_B\parallel \{\parallel S_B(t)-S_B(t_1)\parallel \}.\nonumber \\\le & {} (m_B+\beta _m.1)\{\frac{1}{2}\}+I_B\parallel S_B(t)-S_B(t_1)\parallel .\nonumber \\\le & {} \varphi \parallel S_B(t)-S_B(t_1)\parallel . \end{aligned}$$

Here while we use $\varphi = (m_B+\beta _m)$ with $\psi =1$ and $e=\frac{1}{2}$.

This implies the given function is a bounded function, so we have,

14 $$\begin{aligned} \parallel \Phi _1(t,S_B)- & {} \Phi _1(t,S_{1B})\parallel \le \varphi \parallel S_B(t)-S_B(t_1)\parallel . \end{aligned}$$

Hence, we see that the Lipschitz condition for Eq. (12) is satisfied, also $0\le (m_B+\beta _m\psi )e+I_B<1$, where $\psi =1$ and $e=\frac{1}{2}$, which also emphasized that the said Eq. (12) is contraction. The Lipschitz condition for other equations by the similar way are given below,

15 $$\begin{aligned} \parallel \Phi _2(t,S_p)- & {} \Phi _2(t,S_{1p})\parallel \le \varphi \parallel S_p(t)-S_p(t_1)\parallel .\nonumber \\ \parallel \Phi _3(t,E_p)- & {} \Phi _2(t,E_{1p})\parallel \le \varphi _1\parallel E_p(t)-E_p(t_1)\parallel .\nonumber \\ \parallel \Phi _4(t,I_p)- & {} \Phi _2(t,I_{1p})\parallel \le \varphi _2\parallel I_p(t)-I_p(t_1)\parallel .\nonumber \\ \parallel \Phi _5- & {} \Phi _2(t,A_{1p})\parallel \le \varphi _3\parallel A_p(t)-A_p(t_1)\parallel .\nonumber \\ \parallel \Phi _6(t,R_p)- & {} \Phi _2(t,R_{1p})\parallel \le \varphi _4\parallel R_p(t)-R_p(t_1)\parallel .\nonumber \\ \parallel \Phi _7(t,W)- & {} \Phi _2(t,W)\parallel \le \varphi _5\parallel W(t)-W(t_1)\parallel . \end{aligned}$$

### Theorem 10.2

The Kernals of Eq. (10) $\prod _1, \prod _2$ and $\prod _3$ fulfill the Lipschitz condition and contraction if the following inequality hold. $0\le (\sigma (t)+\alpha \psi )e+I<1$, where $\psi =1$ and $I=e=\frac{1}{2}$.

### Proof

To prove the concern condition suppose that *D* and $D_1$ are any two function then, from $\prod _1$ we write as,

16 $$\begin{aligned} \parallel \prod _1(t,D)-\prod _1(t,D_1)\parallel =\parallel -\sigma (t)SI-\alpha S\parallel . \end{aligned}$$

By triangle inequality Eq. (15) becomes,

17 $$\begin{aligned} \parallel \prod _1(t,D)-\prod _1(t,D_1)\parallel\le & {} \parallel -\sigma (t)SI\parallel +\parallel -\alpha S\parallel .\nonumber \\\le & {} \parallel \sigma (t)SI\parallel +\parallel \alpha S\parallel .\nonumber \\\le & {} \parallel \sigma (t)IS\{D(t)-D_1(t)\}\parallel +\parallel \alpha S\{D(t)-D_1(t)\}\parallel .\nonumber \\\le & {} \parallel \sigma (t)\parallel \parallel I\parallel +\parallel \alpha \parallel \parallel \{D(t)-D_1(t)\}\parallel .\nonumber \\\le & {} \{\sigma (t)+\alpha .1\} \{\frac{1}{2}\}\parallel \{D(t)-D_1(t)\parallel \}.\nonumber \\\le & {} \Omega \parallel \{D(t)-D_1(t)\parallel . \end{aligned}$$

Where $\Omega =\sigma (t)+\alpha$, while again we see that $\psi =1$ and $I=e=\frac{1}{2}$ showing the same effect of disease spread rate through visitors to “Wahan” city or the people living there in “Wahan”.

Hence, Lipschitz condition for Eq. (16) is satisfied, and by similar way we find the remaining Eq. (10) are below, as,

18 $$\begin{aligned} \parallel \prod _1(t,D)- & {} \prod _1(t,D_{1})\parallel \le \Omega _1\parallel E_(t)-E_(t_1)\parallel .\nonumber \\ \parallel \prod _2(t,D)- & {} \prod _2(t,D_{1})\parallel \le \Omega _2\parallel I_(t)-I_(t_1)\parallel . \end{aligned}$$

**Conclusion : 1** In this subsection we conclude that both the population showing the same effect on the new disease **“Corona Virus” ** spread in **China “Wahan”** city. The Eqs. (13), (14), (16), and (17) providing the same values i.e, $\psi =1$ and $e=\frac{1}{2}$, indicated that if the visitors visit the particular city or no visitors there in the concern city the spreading ratio of the disease remain same from the fixed values taken both population. These fixed values of both the population $\psi$ and *e* create a perception that visitors have not a key role in this epidemic disease which spread in particular region of the **“Wahan” city**.

Taking Eqs. (8) and (9) with kernal notation becomes,

19 $$\begin{aligned} S_B(t)= & {} S_B(0)+\frac{2(1-\tau )}{(2-\tau )M(\tau )}\{\Phi _1(t,S_B)\}+\frac{2 \tau }{(2-\tau )M(\tau )} \int ^t_0\{\Phi _1(y,S_B)\}dy,\nonumber \\ S_p(t)= & {} S_p(0)+\frac{2(1-\tau )}{(2-\tau )M(\tau )}\{\Phi _2(t,S_p)\}+\frac{2 \tau }{(2-\tau )M(\tau )} \int ^t_0\{\Phi _2(y,S_p)\}dy,\nonumber \\ E_p(t)= & {} E_p(0)+\frac{2(1-\tau )}{(2-\tau )M(\tau )}\{\Phi _3(t,E_p)\}+\frac{2 \tau }{(2-\tau )M(\tau )} \int ^t_0\{\Phi _3(y,E_p)\}dy,\nonumber \\ I_p(t)= & {} I_p(0)+\frac{2(1-\tau )}{(2-\tau )M(\tau )}\{\Phi _4(t,I_p)\}+\frac{2 \tau }{(2-\tau )M(\tau )} \int ^t_0\{\Phi _4(y,I_p)\}dy,\nonumber \\ A_p(t)= & {} A_p(0)+\frac{2(1-\tau )}{(2-\tau )M(\tau )}\{\Phi _5(t,A_p)\}+\frac{2 \tau }{(2-\tau )M(\tau )} \int ^t_0\{\Phi _5(y,A_p)\}dy,\nonumber \\ R_p(t)= & {} R_p(0)+\frac{2(1-\tau )}{(2-\tau )M(\tau )}\{\Phi _6(t,R_p)\}+\frac{2 \tau }{(2-\tau )M(\tau )} \int ^t_0\{\Phi _6(y,R_p)\}dy,\nonumber \\ W(t)= & {} W(0)+\frac{2(1-\tau )}{(2-\tau )M(\tau )}\{\Phi _7(t,W\sigma )\}+\frac{2 \tau }{(2-\tau )M(\tau )} \int ^t_0\{\Phi _7(y,W)\}dy. \end{aligned}$$

Using technique in Eq. (19), we obtain Eqs. (17) and (18) as Eqs.(20) and (21),

20 $$\begin{aligned} S(t)= & {} S(0)+\frac{2(1-\tau )}{(2-\tau )M(\tau )}\{\prod _1(t,S)\}+\frac{2}{(2-\tau )M(\tau )} \int ^t_0\{\prod _1(y,S)\}dy,\nonumber \\ E(t)= & {} E(0)+\frac{2(1-\tau )}{(2-\tau )M(\tau )}\{\prod _2(t,E)\}+\frac{2}{(2-\tau )M(\tau )} \int ^t_0\{\prod _1(y,E)\}dy,\nonumber \\ I(t)= & {} I(0)+\frac{2(1-\tau )}{(2-\tau )M(\tau )}\{\prod _3(t,I)\}+\frac{2}{(2-\tau )M(\tau )} \int ^t_0\{\prod _1(y,I)\}dy. \end{aligned}$$

Using recurrence formula for Eqs. (19) and (20) we get,

21 $$\begin{aligned} S_{Bn}(t)= & {} \frac{2(1-\tau )}{(2-\tau )M(\tau )}\{\Phi _1(t,S_B(n-1))\}+\frac{2 \tau }{(2-\tau )M(\tau )} \int ^t_0\{\Phi _1(y,S_B(n-1))\}dy,\nonumber \\ S_{pn}(t)= & {} \frac{2(1-\tau )}{(2-\tau )M(\tau )}\{\Phi _2(t,S_p(n-1))\}+\frac{2 \tau }{(2-\tau )M(\tau )} \int ^t_0\{\Phi _1(y,S_p(n-1))\}dy,\nonumber \\ E_{pn}(t)= & {} \frac{2(1-\tau )}{(2-\tau )M(\tau )}\{\Phi _3(t,E_p(n-1))\}+\frac{2 \tau }{(2-\tau )M(\tau )} \int ^t_0\{\Phi _1(y,E_p(n-1))\}d,\nonumber \\ I_{pn}(t)= & {} \frac{2(1-\tau )}{(2-\tau )M(\tau )}\{\Phi _4(t,I_p(n-1))\}+\frac{2 \tau }{(2-\tau )M(\tau )} \int ^t_0\{\Phi _4(y,I_p(n-1))\}dy,\nonumber \\ A_{pn}(t)= & {} \frac{2(1-\tau )}{(2-\tau )M(\tau )}\{\Phi _5(t,A_p(n-1))\}+\frac{2 \tau }{(2-\tau )M(\tau )} \int ^t_0\{\Phi _5(y,A_p(n-1))\}dy,\nonumber \\ R_{pn}(t)= & {} \frac{2(1-\tau )}{(2-\tau )M(\tau )}\{\Phi _6(t,R_p(n-1))\}+\frac{2 \tau }{(2-\tau )M(\tau )} \int ^t_0\{\Phi _6(y,R_p(n-1))\}dy,\nonumber \\ W_n(t)= & {} \frac{2(1-\tau )}{(2-\tau )M(\tau )}\{\Phi _7(t,W(n-1))\}+\frac{2 \tau }{(2-\tau )M(\tau )} \int ^t_0\{\Phi _7(y,W(n-1))\}dy. \end{aligned}$$

And we get also

22 $$\begin{aligned} S_n(t)= & {} \frac{2(1-\tau )}{(2-\tau )M(\tau )}\{\prod _1(t,S(n-1))\}+\frac{2}{(2-\tau )M(\tau )} \int ^t_0\{\prod _1(y,S(n-1))\}dy,\nonumber \\ E_n(t)= & {} \frac{2(1-\tau )}{(2-\tau )M(\tau )}\{\prod _2(t,E(n-1))\}+\frac{2}{(2-\tau )M(\tau )} \int ^t_0\{\prod _1(y,E(n-1))\}dy,\nonumber \\ I_n(t)= & {} \frac{2(1-\tau )}{(2-\tau )M(\tau )}\{\prod _3(t,I(n-1))\}+\frac{2}{(2-\tau )M(\tau )} \int ^t_0\{\prod _3(y,I(n-1))\}dy. \end{aligned}$$

With the initial conditions for Eqs. (21) and (22) are,

$$\begin{aligned} S^0_B(t)=S_B(t),S^0_p(t)=S_p(t),E^0_p(t)=E_p(t),I^0_p(t)=I_p(t), A^0_p(t)=A_p(t),R^0_p(t)=R_p(t),W^0(t)=W(t). \end{aligned}$$

Now to calculate the successive terms, we use the following difference formula,

23 $$\begin{aligned} \Gamma _1n(t)= & {} S_Bn(t)-S_B(n-1)t\nonumber \\= & {} \frac{2(1-\tau )}{(2-\tau )M(\tau )}\{\Phi _1(t,S_B(n-1)-\phi _1(t,S_B(n-2))\}\nonumber \\&+\frac{2 \tau }{(2-\tau )M(\tau )} \int ^t_0\{\Phi _1(y,S_B(n-1)-\phi _1(y,S_B(n-2))\}dy,\nonumber \\ \Gamma _2n(t)= S_pn(t)-S_p(n-1)t= & {} \frac{2(1-\tau )}{(2-\tau )M(\tau )}\{\Phi _2(t,S_p(n-1)-\phi _2(t,S_p(n-2))\}\nonumber \\&+\frac{2 \tau }{(2-\tau )M(\tau )} \int ^t_0\{\Phi _2(y,S_p(n-1)-\phi _2(y,S_p(n-2))\}dy,\nonumber \\ \Gamma _3n(t)= E_pn(t)-E_p(n-1)t= & {} \frac{2(1-\tau )}{(2-\tau )M(\tau )}\{\Phi _3(t,E_p(n-1)-\phi _3(t,E_p(n-2))\}\nonumber \\&+\frac{2 \tau }{(2-\tau )M(\tau )} \int ^t_0\{\Phi _3(y,E_p(n-1)-\phi _3(y,e_p(n-2))\}dy,\nonumber \\ \Gamma _4n(t)= I_pn(t)-I_p(n-1)t= & {} \frac{2(1-\tau )}{(2-\tau )M(\tau )}\{\Phi _4(t,I_p(n-1)-\phi _4(t,I_p(n-2))\}\nonumber \\&+\frac{2 \tau }{(2-\tau )M(\tau )} \int ^t_0\{\Phi _2(y,I_p(n-1)-\phi _4(y,I_p(n-2))\}dy,\nonumber \\ \Gamma _5n(t)= A_pn(t)-A_p(n-1)t= & {} \frac{2(1-\tau )}{(2-\tau )M(\tau )}\{\Phi _5(t,A_p(n-1)-\phi _5(t,A_p(n-2))\}\nonumber \\&+\frac{2 \tau }{(2-\tau )M(\tau )} \int ^t_0\{\Phi _5(y,A_p(n-1)-\phi _5(y,A_p(n-2))\}dy,\nonumber \\ \Gamma _6n(t)= R_pn(t)-R_p(n-1)t= & {} \frac{2(1-\tau )}{(2-\tau )M(\tau )}\{\Phi _6(t,R_p(n-1)-\phi _6(t,R_p(n-2))\}\nonumber \\&+\frac{2 \tau }{(2-\tau )M(\tau )} \int ^t_0\{\Phi _6(y,R_p(n-1)-\phi _6(y,R_p(n-2))\}dy,\nonumber \\ \Gamma _7n(t)= Wn(t)-W(n-1)t= & {} \frac{2(1-\tau )}{(2-\tau )M(\tau )}\{\Phi _7(t,W(n-1)-\phi _7(t,W(n-2))\}\nonumber \\&+\frac{2 \tau }{(2-\tau )M(\tau )} \int ^t_0\{\Phi _7(y,W(n-1)-\phi _7(y,W(n-2))\}dy.\nonumber \\ \end{aligned}$$

Now applying the recurrence formula for visitors population dynamic in Eq. (22) is given by,

24 $$\begin{aligned} \mathfrak {R}_1n(t)= & {} Sn(t)-S(n-1)t\nonumber \\= & {} \frac{2(1-\tau )}{(2-\tau )M(\tau )}\{\prod _1(t,S(n-1)-\prod _1(t,S(n-2))\}\nonumber \\&+\frac{2}{(2-\tau )M(\tau )} \int ^t_0\{\prod _1(y,S(n-1)-\prod _1(y,S(n-2))\}dy,\nonumber \\ \mathfrak {R}_2n(t)= En(t)-E(n-1)t\nonumber \\= & {} \frac{2(1-\tau )}{(2-\tau )M(\tau )}\{\prod _2(t,E(n-1)-\prod _2(t,E(n-2))\}\nonumber \\&+\frac{2}{(2-\tau )M(\tau )} \int ^t_0\{\prod _2(y,E(n-1)-\prod _2(y,E(n-2))\}dy,\nonumber \\ \mathfrak {R}_3n(t)= In(t)-I(n-1)t\nonumber \\= & {} \frac{2(1-\tau )}{(2-\tau )M(\tau )}\{\prod _3(t,S(n-1)-\prod _3(t,I(n-2))\}\nonumber \\&+\frac{2}{(2-\tau )M(\tau )} \int ^t_0\{\prod _3(y,I(n-1)-\prod _3(y,I(n-2))\}dy. \end{aligned}$$

Here we have the following Eq. (25) with $i=1,2,3,4,5,6,7$ and Eq. (26) with $j=1,2,3$ are given,

25 $$\begin{aligned} S_B{n}(t)=\sum ^n_{i=1}\Gamma _1{i}(t), S_p{n}(t)=\sum ^n_{i=1}\Gamma _2{i}(t), E_p{n}(t)=\sum ^n_{i=1}\Gamma _3{i}(t). I_p{n}(t)=\sum ^n_{i=1}\Gamma _4{i}(t),\nonumber \\ A_p{n}(t)=\sum ^n_{i=1}\Gamma _5{i}(t), R_p{n}(t)=\sum ^n_{i=1}\Gamma _6{i}(t), W{n}(t)=\sum ^n_{i=1}\Gamma _7{i}(t). \nonumber \\ \end{aligned}$$

26 $$\begin{aligned} S{n}(t)=\sum ^n_{i=1}\prod _1{i}(t), E{n}(t)=\sum ^n_{i=1}\prod _2{i}(t),\nonumber \\ I{n}(t)=\sum ^n_{i=1}\prod _3{i}(t).\nonumber \\ \end{aligned}$$

Here we use the same strategy and assume the following,

$$\begin{aligned} \parallel \Gamma _1n(t)\parallel= & {} \parallel S_Bn(t)-S_B(n-1)(t)\parallel \\= & {} \parallel \frac{2(1-\tau )}{(2-\tau )M(\tau )}\{\Phi _1(t,S_B(n-1) -\phi _1(t,S_B(n-2))\}\\&\frac{2 \tau }{(2-\tau )M(\tau )} \int ^t_0\{\Phi _1(y,S_B(n-1)-\phi _1(y,S_B(n-2))\}\parallel dy. \end{aligned}$$

Now for above equation we using triangle inequality, we get,

$$\begin{aligned}&\parallel S_Bn(t)-S_B(n-1)(t)\parallel \\&\quad \le \parallel \frac{2(1-\tau )}{(2-\tau )M(\tau )}\{\Phi _1(t,S_B(n-1) -\phi _1(t,S_B(n-2))\}\parallel \\&\quad + \frac{2 \tau }{(2-\tau )M(\tau )} \int ^t_0\parallel \{\Phi _1(y,S_B(n-1)-\phi _1(y,S_B(n-2))\}\parallel dy. \end{aligned}$$

But we have proved that the kernal satisfied the Lipschits criteria then the above becomes,

27 $$\begin{aligned} \parallel Sn(t)-S(n-1)(t)\parallel\le & {} \parallel \frac{2(1-\tau )}{(2-\tau )M(\tau )}\{\Phi _1\parallel \{S_B(n-1)-S_B(n-2))\}\parallel \nonumber \\&+ \frac{2 \tau }{(2-\tau )M(\tau )}\Phi _1 \int ^t_0\parallel \{ S_B(n-1)-S_B(n-2))\}\parallel dy. \nonumber \\ \end{aligned}$$

From simplifying form Eq. (27) implies,

28 $$\begin{aligned} \parallel \Gamma _1n(t)\parallel \le \frac{2(1-\tau )}{2-\tau M(\tau )}\Phi _1\parallel \Gamma _1(n-1)(t)\parallel +\frac{2\tau }{2-\tau (M(\tau ))}\Gamma _1\int ^t_0\parallel \Gamma _1(n-1)y\parallel dy.\nonumber \\ \end{aligned}$$

By the same way we get the following result,

29 $$\begin{aligned} \parallel \Gamma _2n(t)\parallel \le \frac{2(1-\tau )}{2-\tau M(\tau )}\Phi _2\parallel \Gamma _2(n-1)(t)\parallel +\frac{2\tau }{2-\tau (M(\tau ))}\Gamma _2\int ^t_0\parallel \Gamma _2(n-1)y\parallel dy,\nonumber \\ \parallel \Gamma _3n(t)\parallel \le \frac{2(1-\tau )}{2-\tau M(\tau )}\Phi _3\parallel \Gamma _3(n-1)(t)\parallel +\frac{2\tau }{2-\tau (M(\tau ))}\Gamma _3\int ^t_0\parallel \Gamma _3(n-1)y\parallel dy,\nonumber \\ \parallel \Gamma _4n(t)\parallel \le \frac{2(1-\tau )}{2-\tau M(\tau )}\Phi _4\parallel \Gamma _4(n-1)(t)\parallel +\frac{2\tau }{2-\tau (M(\tau ))}\Gamma _4\int ^t_0\parallel \Gamma _4(n-1)y\parallel dy,\nonumber \\ \parallel \Gamma _5n(t)\parallel \le \frac{2(1-\tau )}{2-\tau M(\tau )}\Phi _5\parallel \Gamma _5(n-1)(t)\parallel +\frac{2\tau }{2-\tau (M(\tau ))}\Gamma _5\int ^t_0\parallel \Gamma _5(n-1)y\parallel dy,\nonumber \\ \parallel \Gamma _6n(t)\parallel \le \frac{2(1-\tau )}{2-\tau M(\tau )}\Phi _6\parallel \Gamma _6(n-1)(t)\parallel +\frac{2\tau }{2-\tau (M(\tau ))}\Gamma _6\int ^t_0\parallel \Gamma _6(n-1)y\parallel dy.\nonumber \\ \end{aligned}$$

Similarly from Eq. (24) we have below result,

30 $$\begin{aligned} \parallel \mathfrak {R}_1n(t)\parallel \le \frac{2(1-\tau )}{2-\tau M(\tau )}\prod _1\parallel \mathfrak {R}_1(n-1)(t)\parallel +\frac{2\tau }{2-\tau (M(\tau ))}\mathfrak {R}_1\int ^t_0\parallel \mathfrak {R}_1(n-1)y\parallel dy,\nonumber \\ \parallel \mathfrak {R}_2n(t)\parallel \le \frac{2(1-\tau )}{2-\tau M(\tau )}\prod _2\parallel \mathfrak {R}_2(n-1)(t)\parallel +\frac{2\tau }{2-\tau (M(\tau ))}\mathfrak {R}_2\int ^t_0\parallel \mathfrak {R}_2(n-1)y\parallel dy,\nonumber \\ \parallel \mathfrak {R}_3n(t)\parallel \le \frac{2(1-\tau )}{2-\tau M(\tau )}\prod _3\parallel \mathfrak {R}_3(n-1)(t)\parallel +\frac{2\tau }{2-\tau (M(\tau ))}\mathfrak {R}_3\int ^t_0\parallel \mathfrak {R}_3(n-1)y\parallel dy.\nonumber \\ \end{aligned}$$

Now considered the theorem given below,

### Theorem 10.3

The model defined in system (1) has exact coupled solution if the condition below hold that is we find that

$$\begin{aligned} \frac{2(1-\tau )}{(2-\tau )M(\tau )}\Phi _1+\frac{2\tau )}{(2-\tau )M(\tau )}\Phi _1<1. \end{aligned}$$

### Proof

We shown that all the Eqs. in (28) and (29) are bounded and the functions $S_B, S_p, E_p, I_p, A_p,R_p, W$ fulfill the Lipschitz condition, so Eqs. (28) and (29) by recursive method its succeeding relation are given below,

31 $$\begin{aligned} \parallel \Gamma _1\parallel \le \parallel S_Bn(0)\parallel [\frac{2(1-\tau )}{(2-\tau )M(\tau )}\Phi _1+\frac{2\tau )}{(2-\tau )M(\tau )}\Phi _1]^n,\nonumber \\ \parallel \Gamma _2\parallel \le \parallel S_pn(0)\parallel [\frac{2(1-\tau )}{(2-\tau )M(\tau )}\Phi _2+\frac{2\tau )}{(2-\tau )M(\tau )}\Phi _2]^n,\nonumber \\ \parallel \Gamma _3\parallel \le \parallel E_pn(0)\parallel [\frac{2(1-\tau )}{(2-\tau )M(\tau )}\Phi _3+\frac{2\tau )}{(2-\tau )M(\tau )}\Phi _3]^n,\nonumber \\ \parallel \Gamma _4\parallel \le \parallel I_pn(0)\parallel [\frac{2(1-\tau )}{(2-\tau )M(\tau )}\Phi _4+\frac{2\tau )}{(2-\tau )M(\tau )}\Phi _4]^n,\nonumber \\ \parallel \Gamma _5\parallel \le \parallel A_pn(0)\parallel [\frac{2(1-\tau )}{(2-\tau )M(\tau )}\Phi _5+\frac{2\tau )}{(2-\tau )M(\tau )}\Phi _5]^n,\nonumber \\ \parallel \Gamma _6\parallel \le \parallel R_pn(0)\parallel [\frac{2(1-\tau )}{(2-\tau )M(\tau )}\Phi _6+\frac{2\tau )}{(2-\tau )M(\tau )}\Phi _6]^n,\nonumber \\ \parallel \Gamma _7\parallel \le \parallel Wn(0)\parallel [\frac{2(1-\tau )}{(2-\tau )M(\tau )}\Phi _7+\frac{2\tau )}{(2-\tau )M(\tau )}\Phi _7]^n. \end{aligned}$$

which shows that the existence and as well as the continuity of the concern solutions is valid and proved. Furthermore, to ensure that the above function is a solution of Eq. (3), we proceed as follows:

32 $$\begin{aligned} S_B(t)-S_B(0)=S_Bn(t)-T_1(t),\nonumber \\ S_p(t)-S_p(0)=S_pn(t)-T_2(t),\nonumber \\ E_p(t)-E_p(0)=E_pn(t)-T_3(t),\nonumber \\ I_p(t)-I_p(0)=I_pn(t)-T_4(t),\nonumber \\ A_p(t)-A_p(0)=A_pn(t)-T_5(t),\nonumber \\ R_p(t)-R_p(0)=R_pn(t)-T_6(t),\nonumber \\ W(t)-W(0)=Wn(t)-T_7(t). \end{aligned}$$

Where the terms $T_1(t), T_2(t), T_3(t), T_4(t),T_5(t),T_6(t)$ and $T_7(t)$ are classified as below,

33 $$\begin{aligned} \parallel T_1n(t)\parallel= & {} \parallel \frac{2(1-\tau )}{2-\tau M(\tau )}\Phi _1(t,S_Bn)-\Phi _1(t,S_B(n-1)\nonumber \\&\quad +\frac{2\tau }{2-\tau M(\tau )}\int ^t_0(\Phi _1(y,S_Bn)-\Phi _1(y,S_B(n-1))\parallel dy.\nonumber \\&\qquad \parallel T_1(t)\parallel \le \parallel \frac{2(1-\tau )}{2-\tau M(\tau )}\parallel \Phi _1(t,S_Bn)-\Phi _1(t,S_B(n-1)\parallel \nonumber \\&\quad +\frac{2\tau }{2-\tau M(\tau )}\int ^t_0\parallel (\Phi _1(y,S_Bn)-\Phi _1(y,S_B(n-1))\parallel dy.\nonumber \\\le & {} \frac{2(1-\tau )}{(2-\tau )M(\tau )}\Gamma _1\parallel S_B-S_B(n-1)\parallel +\frac{2\tau }{(2-\tau )M(\tau )}\Gamma _1\parallel S_B-S_B(n-1)\parallel .\nonumber \\ \end{aligned}$$

In recurrence manner we write as

34 $$\begin{aligned} \parallel T_1(t)\parallel \le ((\frac{2(1-\tau )}{2-\tau M(\tau )}+\frac{2\tau }{2-\tau M(\tau )}t_0)^{n+1} \Gamma _1^{n+1}. \end{aligned}$$

Now using limit $n\rightarrow \infty$ on Eq. (34)

$$\begin{aligned} \parallel T_1(t)\parallel \rightarrow 0. \end{aligned}$$

The same procedure using for Eq. (32) we get,

$$\begin{aligned} \parallel T_2(t)\parallel \rightarrow 0,\parallel T_3(t)\parallel \rightarrow 0,\parallel T_4(t)\parallel \rightarrow 0,\\ \parallel T_5(t)\parallel \rightarrow 0,\parallel T_6(t)\parallel \rightarrow 0,\parallel T_7(t)\parallel \rightarrow 0. \end{aligned}$$

To show system (3) having unique solution, we suppose that there exists another solution of system (3) are $S_{1B}(t), S_{1p}(t), E_{1p}(t), I_{1p}(t), A_{1p}(t), R_{1P}(t)$ and $W_{1}(t)$, such that,

35 $$\begin{aligned} S_B(t)-S_{1B}(t)= & {} \frac{2(1-\tau )}{(2-\tau )M(\tau )}\Phi _1(t,S_B)-\Phi _1(t,S_{1B}) \nonumber \\&\quad +\frac{2\tau }{(2-\tau )M(\tau )}\int ^t_0\Phi _1(y,S_B)-\Phi _1(y,S_{1B}) dy. \end{aligned}$$

Now for Eq(34) using $\parallel .\parallel$, and applying Lipschitz condition of kernelwe,

36 $$\begin{aligned}&\parallel S_B(t)-S_{1B}(t)\parallel (1-\frac{2(1-\tau )}{(2-\tau )M(\tau )}\Gamma _1\nonumber \\&\quad -\frac{2\tau }{(2-\tau )M(\tau )}\Gamma _1(t))\le 0. \end{aligned}$$

**Conclusion: 2** The model defined in system (4) by using the strategies of Eqs. (28) and (29), we see that the succeeding relation with continivity like in Eq. (32) the terms $\hat{T}_1$, $\hat{T}_2$, $\hat{T}_3$, showing same behavior of Eq. (34) with assigning new supposition of solution $\hat{S}_1$, $\hat{S}_2$, $\hat{S}_3$, provides us Eq. (36) of the form of Eq. (35) revealed that the spreading of Corona virus in **“Wahan city”** do not effected by visitors (Table 1).

**Table 1:** Parameters Used In BHRP And BRP Visitors Model

Notation	Parameter description	
$n_B$	Bats Birth rate	
$n_H$	Hosts Birth rate	
$n_p$	People Birth rate	
$m_B$	Bats death rate	
$m_H$	hosts death rate	
$1/\omega _B$	Bats incubation period	
$1/\omega _H$	Host incubation period	
$1/\omega _p$	People incubation period	
$1/\hat{\omega }_B$	Latent people period	
$1/\gamma _B$	Bats infection period	
$1/\gamma _H$	Hosts infection period	
$1/\gamma _p$	The symptomatic people infectious period	
$1/\hat{\gamma }_p$	The asymptomatic people infectious period	
$$\beta _B$$	$$I_B $$ to $$S_B$$ transmission rate	
$$\beta _{BH}$$	$$I_B $$ to $$S_H$$ transmission rate	
$$\beta _H$$	$$I_H $$ to $$S_H$$ transmission rate	
$$\beta _p$$	$$I_p $$ to $$S_p$$ transmission rate	
$\beta _W$	*W* to $S_p$ transmission rate	

**Fig. 1:**
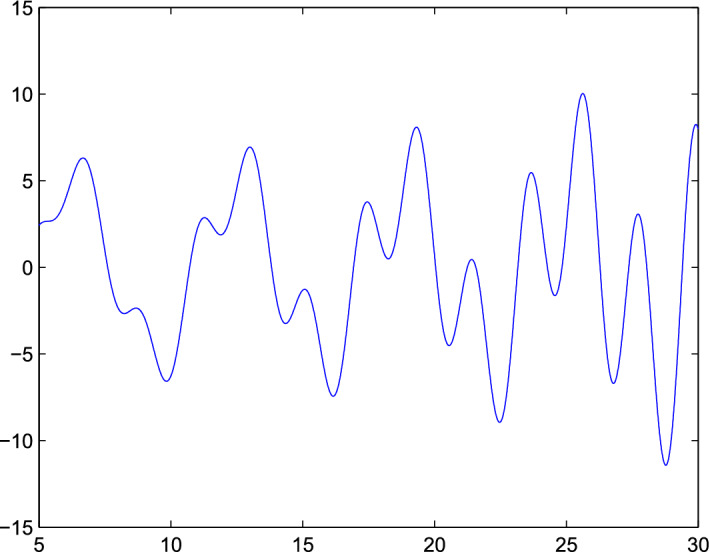
The Plot Show A Random Behavior of Both population model

**Fig. 2:**
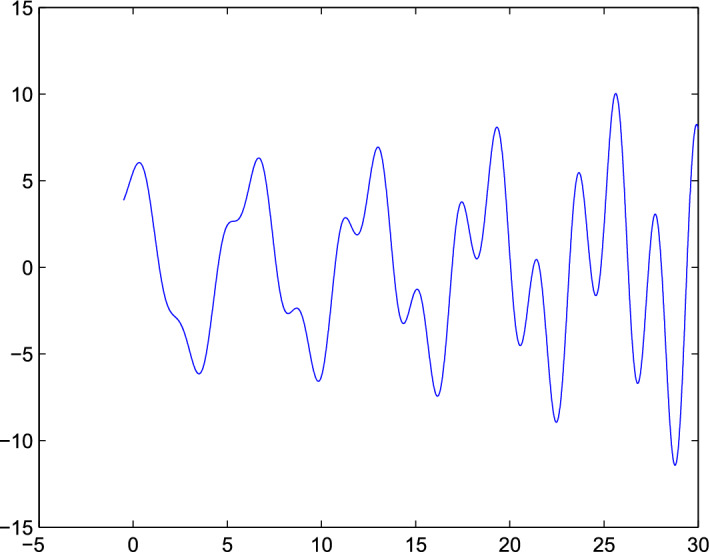
The plot show a random behavior of both population model

**Fig. 3:**
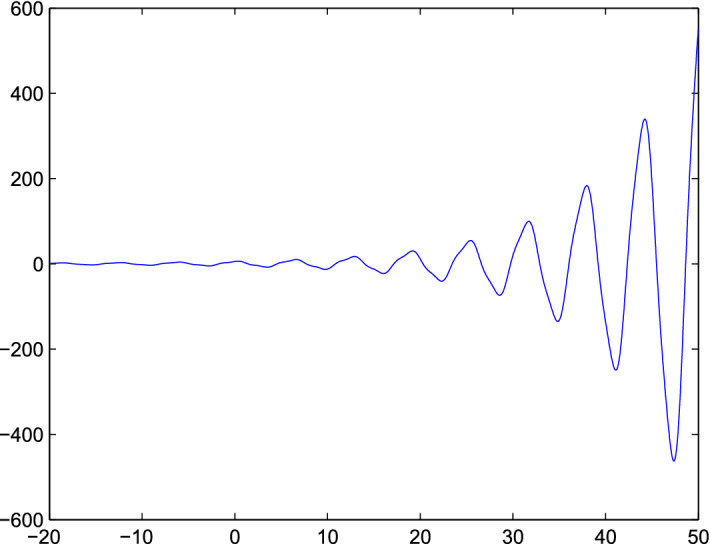
The plot show a random behavior of both population model

### Theorem 10.4

The model (3) solution will unique if

$$\begin{aligned} (1-\frac{2(1-\tau )}{(2-\tau )M(\tau )}\Gamma _1-\frac{2\tau }{(2-\tau )M(\tau )}\Gamma _1 t)>0. \end{aligned}$$

### Proof

If condition defined in above theorem hold then Eq. (36) written as,

37 $$\begin{aligned} \parallel S_B(t)-S_{1B}(t0\parallel =0. \end{aligned}$$

We easily get that,

38 $$\begin{aligned} S_B(t)=S_{1B} \end{aligned}$$

Provided that the following solution of all concern,

$$\begin{aligned} S_p(t)=S_{1p}(t),E_p(t)=E_{1p}(t),I_p(t)=I_{1p}(t),\\ A_p(t)=A_{1p}(t),R_p(t)=R_{1p}(t),W(t)=W{1}(t). \end{aligned}$$

Similarly apply the same theorem (above) and procedure we also find the visitors population as below, provided that the following solution of all concern,

$$\begin{aligned} S(t)=S_1(t),E(t)=E_1(t),I(t)=I_1(t). \end{aligned}$$

## Conclusion

considering the published data with calculating all parameters, we concluded that the models “*BHRP*”, and “*RP*” showed that the spread of Corona virus is very high then MERS in any population. But the addition of our model to published data model showed that the susceptible Bats and visitors to Wuhan or any country having same estimation as that population, more specially visitors of any country are not responsible in the spread of more infection in that area. In simulation from Fig. 1 show that the spread is randomly occurred in your model which shows that, in spread of Corona virus no external agent is involved (visitors). Fig 2 also indicate that the virus may be started from any point where there will be no visitors and susceptible Bats exist. From Fig. 3 we see that the spread of this virus is very fast and then it change in any region of the country. But here our objective of this mathematical model is to estimate the role of susceptible Bats and visitors in spread of “*Corona* $virus''$ in any population. Finally we say that the spread of virus is free from any type of visitors and susceptible Bats.
